# A Structural Model for the Core Nup358-BicD2 Interface

**DOI:** 10.3390/biom13101445

**Published:** 2023-09-26

**Authors:** James M. Gibson, Xiaoxin Zhao, M. Yusuf Ali, Sozanne R. Solmaz, Chunyu Wang

**Affiliations:** 1 Department of Biological Sciences, Department of Chemistry and Chemical Biology, Center for Biotechnology and Interdisciplinary Studies, Rensselaer Polytechnic Institute, 110 8th Street, Troy, NY 12180, USA; 2 Department of Chemistry, Binghamton University, P.O. Box 6000, Binghamton, NY 13902, USA; xzhao55@binghamton.edu; 3 Department of Molecular Physiology and Biophysics, University of Vermont, Burlington, VT 05405, USA; yusuf.ali@med.uvm.edu

**Keywords:** AlphaFold2, intracellular transport, nuclear positioning, Nup358, Bicaudal D2, dynein

## Abstract

Dynein motors facilitate the majority of minus-end-directed transport events on microtubules. The dynein adaptor Bicaudal D2 (BicD2) recruits the dynein machinery to several cellular cargo for transport, including Nup358, which facilitates a nuclear positioning pathway that is essential for the differentiation of distinct brain progenitor cells. Previously, we showed that Nup358 forms a “cargo recognition α-helix” upon binding to BicD2; however, the specifics of the BicD2-Nup358 interface are still not well understood. Here, we used AlphaFold2, complemented by two additional docking programs (HADDOCK and ClusPro) as well as mutagenesis, to show that the Nup358 cargo-recognition α-helix binds to BicD2 between residues 747 and 774 in an anti-parallel manner, forming a helical bundle. We identified two intermolecular salt bridges that are important to stabilize the interface. In addition, we uncovered a secondary interface mediated by an intrinsically disordered region of Nup358 that is directly N-terminal to the cargo-recognition α-helix and binds to BicD2 between residues 774 and 800. This is the same BicD2 domain that binds to the competing cargo adapter Rab6, which is important for the transport of Golgi-derived and secretory vesicles. Our results establish a structural basis for cargo recognition and selection by the dynein adapter BicD2, which facilitates transport pathways that are important for brain development.

## 1. Introduction

**Figure 1:**
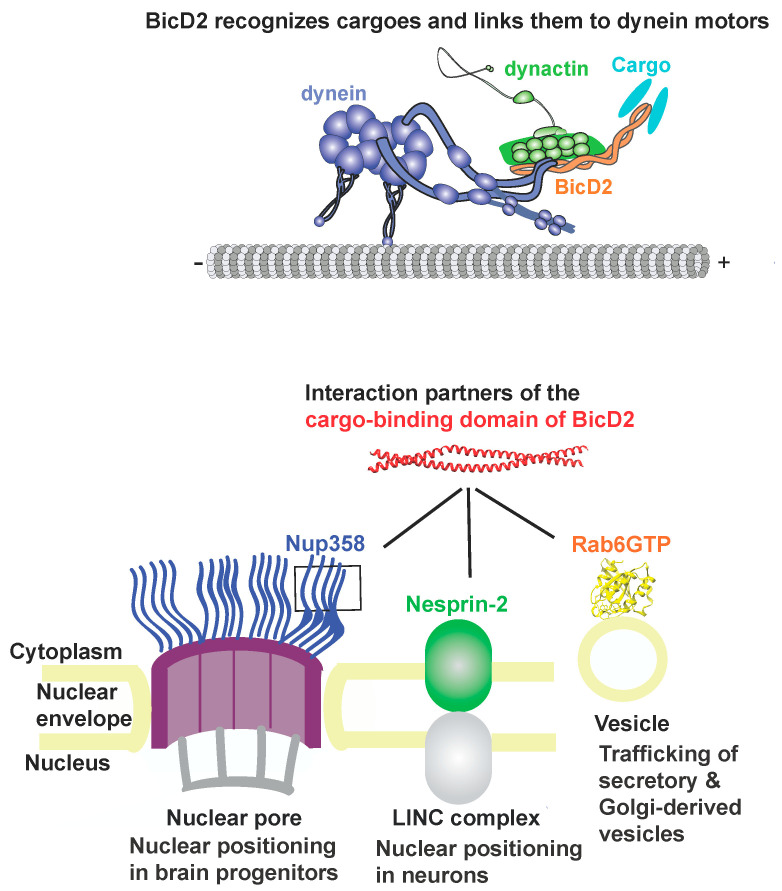
Summary of BicD2/dynein-dependent cellular transport pathways.

Cytoplasmic dynein is the predominant motor responsible for the majority of minus-end-directed traffic on microtubules. Dynein adaptors such as Bicaudal D2 (BicD2) play key roles in the activation and fine-tuning of dynein motility. They recognize cargoes and link them to dynein motors, making them key regulators of cellular transport pathways (Figure 1) [1,2,3,4,5,6,7,8,9,10,11,12,13,14,15]. The dynein adapter BicD2 recruits dynein to several cellular cargoes, including Nup358 (also called RanBP2) [12], the major component of the cytoplasmic filaments of the nuclear pore complex [16], which is embedded in the nuclear envelope. This Nup358/BicD2 pathway for positioning of the cell nucleus is important for initial stages of mitotic spindle assembly in all cells. Furthermore, this pathway is essential for brain development and differentiation of the majority of neurons and glia cells of the neocortex. This is due to the fact that this pathway facilitates apical nuclear migration in radial glial progenitor cells, a nuclear positioning process during brain development that is essential for these brain progenitor cells to enter mitosis and to differentiate [2,9]. BicD2 also recruits dynein to Rab6, a GTPase that is a key regulator of protein secretion and important for the transport of Golgi-derived and secretory vesicles [17,18,19] and to the LINC (linker of nucleoskeleton and cytoskeleton) complex component Nesprin-2, which facilitates a nuclear positioning pathway that is important for neuronal migration of post-mitotic neurons during brain development (Figure 1) [7,20].

The importance of these BicD2-dependent transport pathways is underscored by the fact that mutations of BicD2 cause brain and neuromuscular developmental diseases, including a subset of cases of spinal muscular atrophy, which is the most common genetic cause of death in infants [21,22,23,24,25]. Several of the BicD2 disease mutations cause defects in cargo selection, including increased or decreased affinity to Nup358 and/or Nesprin-2, and/or Rab6, which is associated with defects in apical nuclear migration, neuronal migration, and Golgi morphology [20,21,22]; however, a structural model of a BicD2/cargo complex, which could provide insights into the distinct disease mechanisms of these mutations, is not available. 

BicD2 has three predicted coiled coils (CC1–CC3). Cargo binds to the C-terminal CC3 [26] and structures of the cargo-binding domain have been determined (Figure 1) [27,28,29]. In the absence of cargo, BicD2 is autoinhibited, as the N-terminal dynein-binding site is occluded by the C-terminal cargo-binding domain, forming a looped, closed conformation [27,28,29,30,31]. Binding of cargo results in an open conformation, making BicD2 accessible for dynein binding. Activation of BicD2 is a key step in regulating dynein motility because it is required to activate dynein for processive motility [32,33,34,35]. The stoichiometry of dynein, adaptors, and cargoes in motor complexes is also important for regulation of motility. BicD2 forms dimers and complexes Nup358 with 2:2 stoichiometry [36]. In addition, BicD2 and other dynein adapters also can regulate motility by linking either one or two dynein dimers to cargoes [37]. Surprisingly, it has also been recently established that either one or two dynein adapter dimers such as BicD2 can be recruited to dynein, creating another level of regulation by altering the stoichiometry [38]. Another recurrent theme is the association of dynein with opposite polarity motors such as the plus-end-directed microtubule motor kinesin-1, which binds to the CC2 of BicD2 and to Nup358 and fine tunes overall motility for the Nup358/BicD2 transport pathway [12,18]. The same domain of Nup358 that binds to BicD2 (Nup358-min) also has a LEWD sequence motif, which acts as a binding site for kinesin-1 [12,13,39,40,41]. The LEWD motif is close to the BicD2/dynein-binding site (~30 residues away) but does not overlap with it [42]. Thus, adapter proteins such as BicD2 and Nup358 have key roles in modulating motility, but the underlying mechanism is not well understood since a structure of a BicD2/cargo complex is currently not available.

Previously, we were able to show that Nup358-min, which is the minimal domain that binds both BicD2 and kinesin-1, undergoes a partial transition from a random coil to an α-helix upon binding to BicD2-CTD [42]. Mutations in this Nup358 α-helix mutations result in impaired motility of assembled dynein/dynactin/BicD2/Nup358 complexes, highlighting the important role of the cargo-recognition α-helix and its interactions with BicD2 in activation of dynein for processive motility [42]. Open questions remained regarding the binding region of BicD2 and the details of the Nup358-min/BicD2-CTD interface. The structure of a BicD2/cargo complex is important to provide insights into how these interactions regulate dynein and kinesin-1 motility. 

With the recent development of AlphaFold2 [43,44], we have the advantage of using a highly accurate protein structure prediction package to give us insight into the likely binding sites on both sides of the Nup358/BicD2 interaction, which is in line with our prior results. We also validated the structural model by mutagenesis and other docking packages [45,46,47,48]. Our data predict that the Nup358 cargo-recognition α-helix binds in antiparallel orientation to the center of the BicD2-CTD (residues 747–772), and two salt bridges play crucial roles in the interaction. We also identified a second, weaker interface between the N-terminal intrinsically disordered residues of Nup358-min (2148–2158) and the C-terminal BicD2 region (residues 773–804). This region of Nup358 binds to the same site on BicD2 as Rab6, explaining the previous observation that Rab6 and Nup358 compete for binding to BicD2 [36].

## 2. Materials and Methods

### 2.1. GST Pulldown Assays 

All expression constructs and point mutants were created by a cloning service and codon optimized for expression in *E. coli* (Genscript, Piscataway, NJ, USA).

The expression constructs for His_6_-tagged human BicD2-CTD (residues 715–804, NCBI database entry NM_029791.4), and GST-tagged human Nup358-min (residues 2148–2240, NCBI database entry XM_005264002.2) were previously described [28,42]. Human Rab6a/Q72L (NCBI BC096818.1) was cloned into the pGEX6P1 vector with the BamHI and XhoI restriction sites, which resulted in the expression of a GST-fusion protein. The Q72L mutation locks it in the GTP-bound state, rendering it GTPase-deficient [17]. We refer to Rab6a/Q72L as Rab6^GTP^ throughout the text. 

Rab6^GTP^, Nup358-min, and BicD2-CTD were expressed in the *E. coli* BL20(DE3)-RIL strain at 37 °C as previously described [49]. GST-pull down assays of GST-tagged Nup358-min, GST-tagged Rab6^GTP^, and His_6_-tagged BicD2-CTD were performed as described [42]. For the GST-pulldown, *Hs* BicD2 CTD WT and mutants were purified as described by a single affinity chromatography step from 1 L of cell culture. Nup358-GST constructs (aa 2148–2240 (Nup358-min), aa 2148–2188 and aa 2148–2199) and Rab6^GTP^ were purified from 0.5 L of cell culture by glutathione sepharose, but not eluted, and washed. For Rab6, 1 mM GTP and 2 mM MgCl_2_ were added and incubated for 30 min. Purified BicD2-CTD was added to the column with the bound Nup358 fragments or Rab6 and incubated for 30 min. Columns were washed and eluted with glutathione elution buffer as described [42]. The elution fractions were analyzed by SDS-PAGE (16% acrylamide gels) and stained with Coomassie blue. 

Protein concentrations were determined by spectrophotometry using the peptide bond method. The gel band intensities were quantified using Image J 1.52v as described [20,42,50]. For quantifying the ratio of Nup358 fragments (residues 2148–2240, 2148–2188, 2148–2199) bound to BicD2-CTD from pulldowns, Nup358-min and BicD2-CTD intensities were converted to the protein concentration (mg/mL) using a band of 2 µg of each Nup358 fragment on the same gel as the standard. The concentrations were divided by the molar masses of the fragments to convert them to molar concentrations. Statistical analysis was conducted with the PRISM 9 program (Graphpad, Boston, MA, USA).

### 2.2. AlphaFold2

For AlphaFold2 [43,44], we used the multimer mode in AlphaFold2 Colab (https://colab.research.google.com/github/deepmind/alphafold/blob/main/notebooks/AlphaFold.ipynb), accessed on 12 December 2022 and predicted a complex with two molecules of BicD2-CTD and two molecules of Nup358-min. It had been previously shown that BicD2-CTD and Nup358-min form a 2:2 complex [36].

To generate additional structural models, we also used ColabFold [51]. This Colab notebook was accessed with the following link: https://colab.research.google.com/github/sokrypton/ColabFold/blob/main/AlphaFold2.ipynb on 1 August 2023. The results from the ColabFold run are shown in Figure S1 in the supporting information.

### 2.3. ClusPro

For ClusPro [45,46,52,53], we used the coordinates of the X-ray structure of the human BicD2-CTD dimer at 2.0 Angstrom resolution (PDB ID 6OFP, https://doi.org/10.2210/pdb6OFP/pdb [28]), and then we added one Nup358 entity, reduced to the minimal helical portion (from R2162 to L2184) [42]. A new run was performed with the extended helical region determined from AlphaFold2 (T2160 to N2186). The highest scoring model from ClusPro was then analyzed in MOE (Molecular Operating Environment (MOE), 2022.02 Chemical Computing Group ULC, 910-1010 Sherbrooke St. W., Montreal, QC H3A 2R7, Canada, 2023), and this was then compared to the model from AlphaFold2.

### 2.4. HADDOCK

For HADDOCK [47,48,54], the strategy was similar to the ClusPro strategy. Thus, we used the coordinates of the X-ray structure of the human BicD2-CTD dimer at 2.0 Angstrom resolution (PDB ID 6OFP, https://doi.org/10.2210/pdb6OFP/pdb) [28] and then we added one Nup358 entity, reduced to the minimal helical portion (R2162 to L2184) [42]. However, unlike AlphaFold2, which only uses the sequence information, and ClusPro, which only uses the PDB information, HADDOCK requires some a priori docking sites to be entered. While we have enough experimental data to correctly identify the Nup358 docking sites, the BicD2 side remained significantly underdetermined, which means we could not only use mutagenesis results to input the BicD2 docking sites from HADDOCK. Therefore, the sites with the salt bridges or hydrogen bonds were selected based on the AlphaFold2 results, while the remaining sites were set as neither active nor passive sites.

### 2.5. Alignment of Computational Results

The results from HADDOCK and ClusPro were aligned against the results from AlphaFold2 using MOE [54]. Using only one strand of BicD2 and one strand of Nup358, the residues D755, E756, D762, and E763 in BicD2 were aligned in all cases. Overall measurements of RMSD were determined from the superposition of both protein strands.

## 3. Results

**Figure 2:**
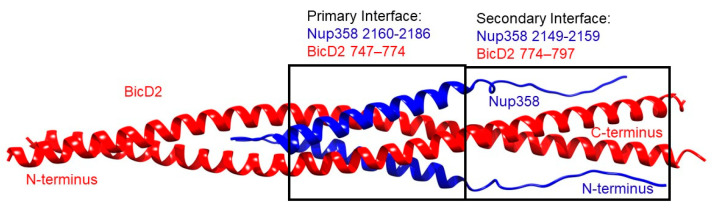
**Nup358/BicD2 interface from AlphaFold2.** In the primary binding site #1, the α-helical region in Nup358 (blue) forms an α-helical bundle with the homo-dimeric coiled-coil of BicD2 (red). This is the high-confidence binding site as scored by AlphaFold2. A secondary binding site is observed in the random-coil region of Nup358, toward the N-terminus of Nup358-min and the C-terminus of the BicD2-CTD. This binding site has a lower confidence score in AlphaFold2 but is supported by our NMR and mutagenesis results. See Table S1.

We obtained a structural model for the 2:2 Nup358-min/BicD2-CTD complex from AlphaFold2, which includes the minimal Nup358 domain that binds both kinesin-1 and BicD2 (Nup358-min, residues 2148–2240) and the minimal cargo-binding domain of BicD2 (BicD2-CTD, residues 715–804) (Figure 2).

**Figure 3:**
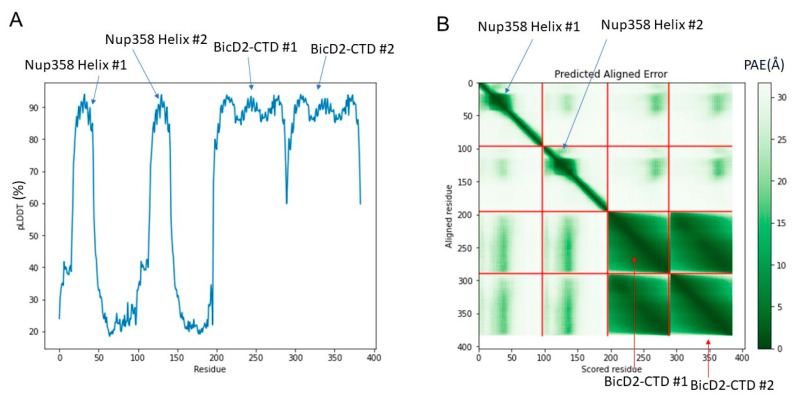
**Predicted per-residue local distance difference test pLDDT and Predicted Aligned Error (PAE) plots for the highest ranked AlphaFold2 model**. (**A**) pLDDT plot. A score of above 80% suggests confidence in the structure prediction (90–100%—high confidence; 80%—confidence threshold; 50%—low confidence). (**B**) PAE plot. A color-gradient indicates the PAE in Å (0–10 Å—low error; 15 Å—acceptable threshold; 30 Å—low confidence).

The structure is composed of a high confidence interface region between α-helical BicD2 and the cargo-recognition α-helix of Nup358, a high confidence non-binding region of BicD2, a low confidence interface region of the N-terminal intrinsically disordered Nup358-min domain immediately adjacent to the cargo-recognition α-helix, and a low-confidence non-binding region that is intrinsically disordered in Nup358. The overall confidence results from AlphaFold2 are shown in Figure 3. The per-residue local distance difference test (pLDDT) score of the BicD2-CTD and the Nup358 cargo-recognition α-helix that interacts with it is between 80–96% (with exception of a few residues at the N- and C-termini), indicating a high confidence in the structure prediction. The pLDDT score of the N-terminal intrinsically disordered Nup358-min region that forms the second interacting site is 30–40%, thus the confidence is low compared to the rest of the complex. The predicted aligned error (PAE), which assesses the confidence in the respective placement of the interaction partners, is for the BicD2-CTD and the Nup358 cargo-recognition α-helix between 0 and 15 Å, which indicates confidence in the prediction. The PAE score for the intrinsically disordered Nup358 region that forms the second interacting site is 20–30 Å, suggesting a lower confidence in that portion of the model. We also performed Alphafold2 predictions with larger domains of Nup358 and BicD2, including the intact CC3 of BicD2. The predicted Nup358-min/BicD2-CTD interface of the larger complex was very similar to the minimal complex shown in Figure 1, however, the additional regions of the larger complex had low confidence scores (Figure S2), which is why we focused on the minimal complex.

The focus of this manuscript is on the high-confidence α-helical interface region of Nup358-min that interacts with BicD2-CTD (Figure 2 and Figure 3). In addition, we include the adjacent intrinsically disordered region of Nup358 that also is predicted to bind to BicD2 but has a lower confidence score, as it is supported by NMR and mutagenesis. The interaction of the cargo-recognition α-helix of Nup358 as well as of the intrinsically disordered N-terminal part of Nup358-min with BicD2 is supported by our published NMR titration results of ^15^N-labeled Nup358-min with BicD2-CTD [42]. Intriguingly, the heights of the peaks corresponding to the Nup358 cargo-recognition α-helix, as well as the adjacent N-terminal intrinsically disordered Nup358 residues 2148–2158, which are predicted to bind to BicD2 in the Alphafold2 model, were significantly reduced in the HSQC spectrum of the Nup358/BicD2 complex compared to the apo state, indicating that this region was important for the interaction between Nup358-min and BicD2-CTD [42]. Figure 2 shows the interface region of the predicted Nup358-min/BicD2-CTD complex. For Nup358-min, this encompasses the 27-residue α-helical region from aa 2160–2186, as well as the immediately preceding 12 residues from aa 2149 to 2159 (Table S1). For BicD2, this interface region is shown to encompass the α-helical region from aa 739 to 797 (Table S1). It should be noted that the two proteins interact in an anti-parallel manner. While the specific interacting residues from Nup358 agree with our prior publication [42], the information involving the BicD2 interacting residues is new. 

### 3.1. The C-Terminal Residues 2189–2240 of Nup358-min Do Not Engage in the Interaction with the BicD2-CTD 

**Figure 4:**
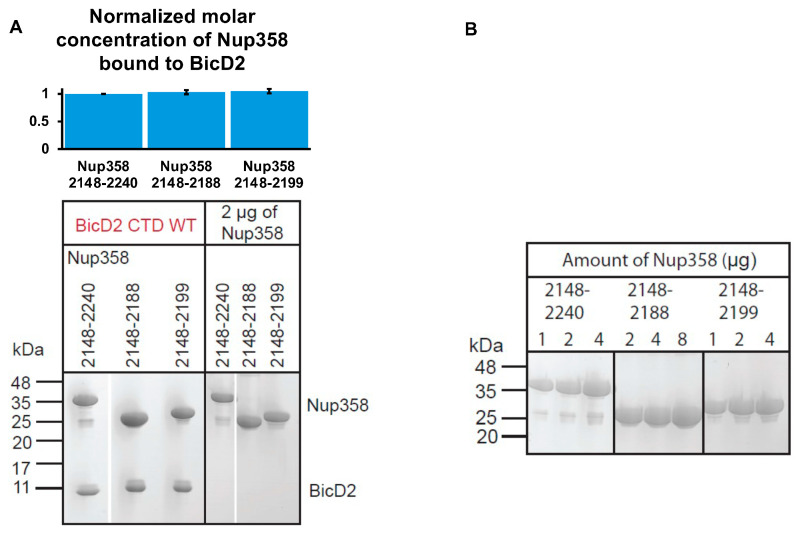
**The C-terminal residues 2189–2240 of Nup358-min do not engage in the interaction with the BicD2-CTD.** (**A**) Bottom panel: Coomassie-stained SDS-PAGE gel of elution fractions of the pulldown of GST-tagged Nup358-min and two C-terminal deletion constructs with the BicD2-CTD. Amounts of 2 µg of each of the three Nup358 fragments were analyzed on the same gel for calibration purposes. The molar masses of standards are indicated on the left. Top panel: The bar graph shows the molar ratio of Nup358 bound to BicD2. This ratio was obtained by quantification of the protein bands of the SDS-PAGEs of the elution fractions of the pull-down experiments. A representative dataset is shown in (**A**). The average ratio from 3 sets of experiments is shown, and the error bars show the standard deviation. The intensities of the gel bands were first converted to concentrations using the 2 µg bands of each Nup358 fragment as a standard. To obtain molar ratios, the concentrations were divided by the molar mass of the Nup358 fragments. Ratios of the deletion fragments were normalized to the ratio of Nup358-min/BicD2-CTD (=1). (**B**) Different amounts of the three Nup358 fragments were analyzed on SDS-PAGE and stained by Coomassie blue. Statistical analysis: The ratios of bound BicD2 to the three Nup358 fragments (aa 2148–2240 (Nup358-min), aa 2148–2188, and aa 2148–2199) are not statistically significantly different. The statistical significance of the results was assessed using the one-way ANOVA test (two-case multiple comparison). *p* values of less than 0.05 signify significant differences. *p* value for Nup358-min vs. Nup358 2148–2188: 0.61. *p* value for Nup358-min vs. Nup358 2148–2199: 0.33. Original Coomassie-stained SDS-PAGE images can be found in supplementary materials.

We have previously published NMR titration results of ^15^N labeled Nup358-min which was titrated with the BicD2-CTD [42]. For the Nup358-min region that is C-terminal of the cargo-recognition α-helix (aa 2192–2240), no significant changes of the peak height are observed between the HSQCs of apo Nup358-min and the Nup358-min/BicD2-CTD complex, indicating that this region does not engage in the interaction. To confirm this, we assessed the interaction by pull-down assays of GST-tagged Nup358-min (aa 2148–2240) and two C-terminal truncation fragments (aa 2148–2188 and aa 2148–2199) with BicD2-CTD. For all three Nup358 fragments, similar molar ratios of the BicD2-CTD were coeluted (Figure 4). These results confirm that residues aa 2189–2240 of Nup358 do not engage in the interaction with the BicD2-CTD. It should be noted that this region contains the LEWD amino acid motif that acts as kinesin-1 recruitment site [39].

### 3.2. The Interface between the Cargo-recognition α-Helix of Nup358 and the BicD2-CTD

**Figure 5:**
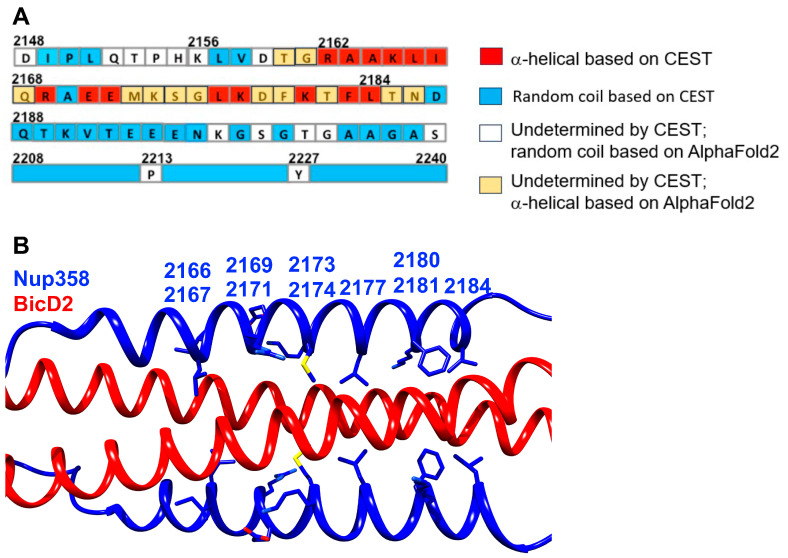
**Nup358/BicD2 interface determined by NMR and Alphafold2.** (**A**) Prior results assigned random coil (blue), α-helical (red), and undetermined (white) conformations to each residue of the Nup358-min sequence bound to BicD2 [42]. Many of the experimentally undetermined amino acids were shown to be in the α-helical region based on AlphaFold2, as shown in orange. (**B**) Cartoon representation of the structural model of the minimal Nup358/BicD2 complex, zoomed in on the interaction of the Nup358 cargo-recognition α-helix with BicD2. The Nup358 residues that were identified as interface residues by mutagenesis and pull-down assays in our prior publication [42] are shown in stick representation and labelled. Note that they also mediate interactions in the Alphafold2 model of the complex.

CEST NMR results from our prior publication suggested that the α-helical binding region of Nup358-min consisted of 23 residues from R2162 to L2184 [42]. However, the data were ambiguous for D2159, T2160, and G2161 towards the N-terminus and T2185 and N2186 towards the C-terminus. The results from AlphaFold2 suggest that T2160, G2161, T2185, and N2186 are part of the α-helix, which is consistent with the previous results, but fills in knowledge gaps. The combined results of the prior publication and the α-helical region of Nup-358 based on the AlphaFold2 model are shown in Figure 5. Table 1 shows a comparison of the Nup358 results from AlphaFold2 to the NMR titration and CEST NMR results from our prior publication [42].

In terms of the Nup358 residues that bind BicD2 according to AlphaFold2, we found similar results compared to our prior publication [42]. The following residues were previously noted as interacting residues: L2166, I2167, R2169, E2171, M2173, K2174, L2177, F2180, K2181, and L2184. All ten of them are also at the interface in the AlphaFold2 model (Table S1 and Figure 5B). 

**Figure 6:**
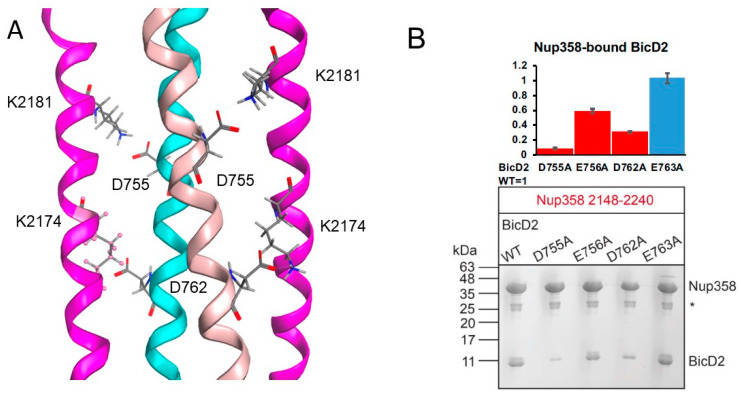
**Two critical interfacial salt bridges predicted by AlphaFold2 and confirmed by mutagenesis**. (**A**) Structure of the minimal Nup358/BicD2 complex in cartoon representation (Nup358 molecules in pink; BicD2 molecules in cyan and light pink). Close-up of salt bridges formed by BicD2 residue D755 and Nup358 residue K2181, as well as BicD2 residue D762 and Nup358 residue K2174. The complex is a symmetric hetero-tetramer. (**B**) The importance of these salt bridges was confirmed by mutagenesis. The bar graph shows the ratio of bound BicD2/Nup358 from pulldown assays normalized respective to the WT (WT = 1; statistically significantly reduced binding = red; normal binding = blue). Average ratios are shown and the error bars represent the standard deviation from three experiments. A representative Coomassie-stained SDS-PAGE of the elution fractions of the pull-downs of GST-tagged Nup358-min with BicD2-CTD WT and distinct BicD2 mutants targeting the highlighted salt bridges is shown. The staining intensities of the gel bands for Nup358 and BicD2 were quantified in ImageJ 1.52v and the background intensity was subtracted to calculate the ratios of bound BicD2/Nup358 which were normalized respective to the ratio obtained for the WT. The positions of molecular weight standards are indicated on the left. An asterisk indicates the location of the GST band. Mutations of the salt bridge residues, D775A, and D762A strongly diminish binding to Nup358-min; meanwhile, the mutations of nearby residues E756A and E763A, not involved in predicted salt bridges, have little effect. The MW standards are indicated on the left. Statistical analysis: The statistical significance of the results was assessed using the one-way ANOVA test (two-case multiple comparison). *p* values of less than 0.05 signify significant differences. The ratios of bound BicD2/Nup358 were compared for the WT with each mutant and *p* values were calculated. *p* values: D755A < 0.0001; E756A < 0.0001; D762A < 0.0001; E763A = 0.8506 (ns).

Based on the AlphaFold2 model, two important salt bridges were identified—these are the salt bridges between Nup358 residue K2174 and BicD2 residue D762, as well as between Nup358 residue K2181 and BicD2 residue D755. We tested the importance of these two salt bridges using mutagenesis (Figure 6A). BicD2 residues D755 and D762 were mutated to alanine, and binding of the mutants to GST-tagged Nup358-min was assessed by pull-down assays. Binding is strongly reduced for both mutants (see quantification in Figure 6B), which agrees with the models obtained from AlphaFold2 and docking programs, supporting the idea that these form a salt bridge. Our prior results in Gibson et al. [42] also showed that K2174A and K2181A mutations in Nup358 greatly reduce BicD2 binding. Here, in Figure 6, we show that D755A and D762K greatly reduce BicD2 binding in pulldown assays, while a lesser effect was observed for E756A or E763A (see quantification in Figure 6B). Thus, the new mutagenesis data for BicD2 residues D755 and D762 support the two critical salt bridges observed from AlphaFold2, ClusPro, and HADDOCK.

**Figure 7:**
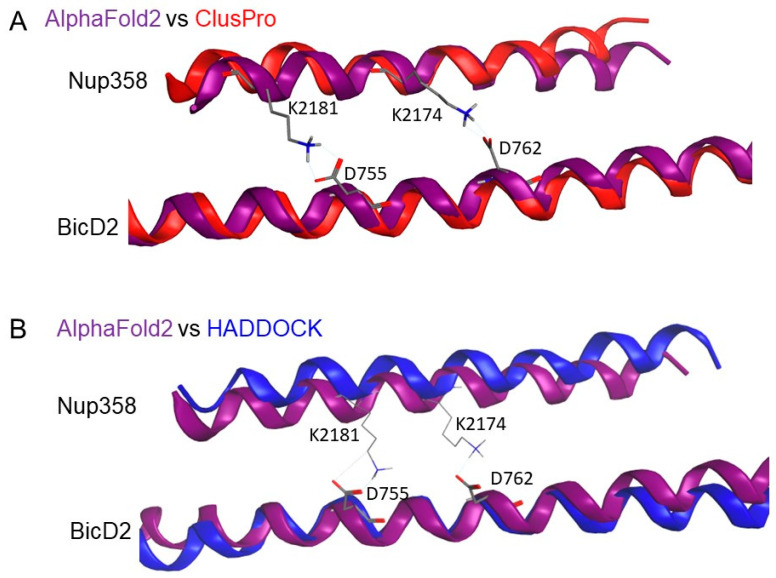
**Nup358/BicD2 interfaces from ClusPro and HADDOCK are similar to those generated by AlphaFold2.** Overlays of AlphaFold2 and (**A**) ClusPro and (**B**) HADDOCK models are showing nearly identical placement of the Nup358 α-helix compared to the set position of the BicD2 dimer in the binding region. Salt bridges identified by ClusPro and HADDOCK, which are identical in position to those from AlphaFold2, are shown in the figure. Alignment using MOE resulted in the following RMSD values: 0.34 and 1.1 Å for the AlphaFold2/ClusPro comparison of BicD2 and Nup358, respectively, and 0.95 and 0.74 Å for the AlphaFold2/HADDOCK comparison of BicD2 and Nup358, respectively.

To further validate the model, we used two rigid-body docking programs, HADDOCK [47,48] and ClusPro [45,46,52,53], to model the structure of the Nup358-min/BicD2-CTD complex. Note that AlphaFold2 used the entire Nup358-min and BicD2-CTD sequence, while ClusPro and HADDOCK used rigid-body models that only included the helical section of Nup358-min together with the X-ray structure of the BicD2-CTD [28]. The resulting models of the complex from HADDOCK and ClusPro replicate many of the key interactions predicted using AlphaFold2. In particular, the highest scoring ClusPro and HADDOCK models also included the salt bridges formed by residue K2181 and D755 and by residues K2174 and D762, which are explicitly shown in Figure 7. Overall, the two programs place the Nup358 domain in very similar locations of BicD2, with only a little bit of variability towards the termini of the Nup358 α-helix. Figure 7 shows the overlay of a single molecule of BicD2 and a single molecule of Nup358-min from AlphaFold2 with the leading ClusPro and HADDOCK models. After alignment, an overall RMSD was calculated for the Nup358-min and BicD2-CTD. The RMSD values were between 0.3 and 1.1 Å and are shown in Table 2. For more information on the scoring of the ClusPro and the HADDOCK models, please see the supplementary information.

While the α-helical region of Nup358, which is shown to bind to BicD2 in all three docking programs, contains several salt bridges and hydrogen bonds, AlphaFold2 suggests that hydrophobic interactions may be an important component in the extended portion of Nup358-min toward the N-terminus.

We conclude that the programs Alphafold2, HADDOCK, and ClusPro predict almost identical structures to those of RMSD of less than 1.2 Å for the Nup358-min/BicD2-CTD complex. Salt bridges formed between BicD2 residue D755 and Nup358 residue K2181 and between BicD2 residue D762 and Nup358 residue K2174 are important to stabilize the interface and are predicted by all three programs. For a list of all interacting residues based on AlphaFold2, please see the supporting information.

### 3.3. A Second Interface Was Identified between Intrinsically Disordered Residues 2149–2159 of Nup358 and the C-Terminal Region of the BicD2-CTD

**Figure 8:**
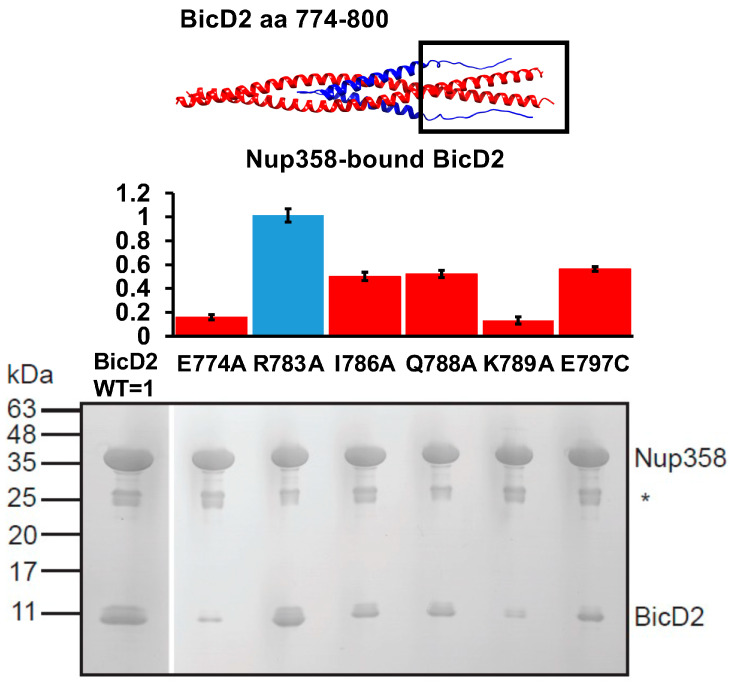
A second interface was identified between intrinsically disordered residues 2148–2158 of Nup358 and the C-terminal region of the BicD2-CTD. Top panel: cartoon representation of the Alphafold2 model of the Nup358-min/BicD2-CTD complex (Nup358 blue, BicD2 red). The interfacial region being studied is boxed. Middle panel: Bar graph showing the ratio of bound BicD2/Nup358 from pulldown assays normalized respective to the WT (WT = 1; statistically significantly reduced binding = red; normal binding = blue). Ratios were presented as average from three experiments and the error bars show the standard deviation from three experiments. Bottom panel: Coomassie-stained SDS-PAGE of elution fractions of GST-pulldowns of GST-tagged Nup358-min with BicD2-CTD WT and mutants. The gel band intensities were quantified to obtain the ratios shown in the bar graph. MW standards are indicated on the left. An asterisk indicates the location of the GST band. Statistical analysis: The statistical significance of the results was assessed using the one-way ANOVA test (two case multiple comparison). *p* values of less than 0.05 signify significant differences. The ratios of bound BicD2/Nup358 were compared for the WT with each mutant and *p* values were calculated: *p* values: E774A < 0.0001; R783A = 0.9993 (ns); I786A < 0.0001; Q788A < 0.0001; K789A < 0.0001; E797C < 0.0001.

Intriguingly, we identified a second interface in the Alphafold2 model of the Nup358/BicD2 complex in addition to the one formed by the Nup358 cargo-recognition α-helix. This interface is formed by the intrinsically disordered residues 2149–2159 of Nup358 which are the N-terminal of the cargo-recognition α-helix. These residues interact with the extreme C-terminal domain of the BicD2-CTD (Figure 8). 

This is in line with previously published NMR titration results of ^15^N-labeled Nup358-min, which was titrated with BicD2-CTD [42]. Intriguingly, the heights of the peaks corresponding to Nup358 residues 2149–2159 were significantly reduced in the HSQC spectrum of the Nup358/BicD2 complex compared to the apo state, indicating that this region was important for the interaction between Nup358-min and BicD2-CTD. While no CEST-positive residues were identified in this region, there were several residues in this region that could not be assessed by CEST. 

Based on the Alphafold2 model, we designed mutants of BicD2-CTD that interacted with residues 2149–2159 of Nup358 and evaluated the interaction of these mutants with GST-tagged Nup358-min by pulldown assays (Figure 8). The ratio of coeluted BicD2-CTD to Nup358-min was quantified. BicD2-CTD mutants E774A, I786A, Q788A, K789A, and E797C diminished binding to Nup358, while the mutant R783A did not diminish the interaction. These results confirm that the intrinsically disordered residues 2148–2158 of Nup358, which are N-terminal to the cargo-recognition α-helix of Nup358, are important for the interaction with BicD2-CTD. These residues interact with the C-terminal region of the BicD2-CTD while the cargo-recognition α-helix binds in the center of the BicD2-CTD. These results also support an anti-parallel configuration of the Nup358-min/BicD2-CTD complex. 

### 3.4. BicD2 Residues 745–763 Bind to Nup358 but Not to Rab6

BicD2 recognizes multiple cargoes, including Rab6^GTP^ which is a key regulator for protein secretion and important for the transport of Golgi-derived and secretory vesicles [17,18,19]. The binding site of Rab6^GTP^ has been previously mapped to residues 755–802 of BicD2-CTD, while the minimal region required for Nup358 binding was mapped to a larger area that included residues 724–802 [27,29]. We also previously established that the Rab6^GTP^-interacting domain on BicD2 is located in an area that is not expected to undergo coiled-coil registry shifts, whereas the Nup358-binding domain is located in an area that is expected to undergo coiled-coil registry shifts. Interestingly, the registry-locked F743I/R747C mutation, a combination of two mutants that caused coiled-coil registry shifts in MD simulations [28,49], displayed normal binding to Rab6^GTP^, while binding to Nup358-min was greatly reduced [49]. Based on these results, we proposed that a registry shift in BicD2-CTD could result in changes of cargo selectivity. 

**Figure 9:**
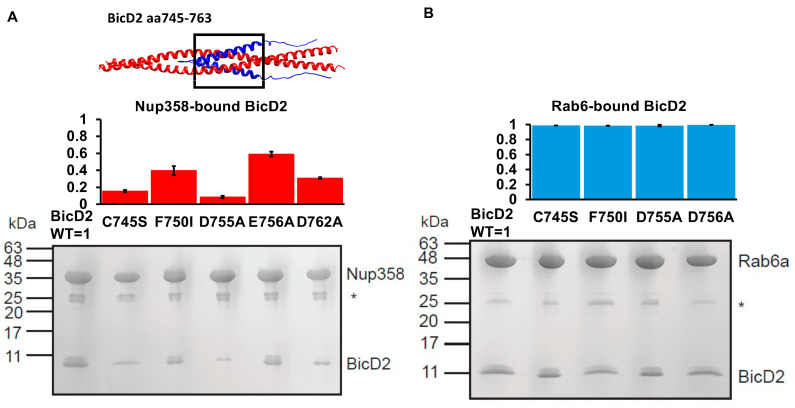
**Nup358 and Rab6 bind to distinct sites on BicD2 CTD.** (**A**) Top panel: cartoon representation of the Alphafold2 model of the Nup358-min/BicD2-CTD complex (Nup358 blue, BicD2 red). The interface being studied is boxed. Middle panel: bar graph showing the ratio of bound BicD2/Nup358 from pulldown assays normalized respective to the WT (WT = 1; statistically significant reduced binding = red; normal binding = blue). Bottom panel: Coomassie-stained SDS-PAGE of elution fractions of GST-pulldowns of GST-tagged Nup358-min with BicD2-CTD WT and mutants. An asterisk indicates the location of the GST band. The gel band intensities were quantified to obtain the ratios shown in the bar graph. (**B**) Top panel: bar graph showing the ratio of bound BicD2/Rab6^GTP^ from pulldown assays normalized respective to the WT. Bottom panel: Coomassie-stained SDS-PAGE of elution fractions of GST-pulldowns of GST-tagged Rab6 with BicD2-CTD WT and mutants. (**A**,**B**) Ratios were averaged from three experiments; the error bars show the standard deviation. Molar masses of standards are indicated on the left. Statistical analysis: The statistical significance of the results was assessed using the one-way ANOVA test (two-case multiple comparison). *p* values of less than 0.05 signify significant differences. The ratios of bound BicD2/Nup358 or BicD2/Rab6 were compared for the WT with each mutant and *p* values were calculated. *p* values: C745S < 0.0001; F750I < 0.0001; D755A < 0.0001; E756A < 0.0001; D762A < 0.0001. C745S = 0.80 (ns); F750I = 0.64 (ns); D755A = 0.75 (ns); E756A = 0.997 (ns).

In the AlphaFold2 model of the Nup358-min/BicD2-CTD complex, the cargo-recognition α-helix of Nup358 binds to residues 747–774 of BicD2-CTD. We mutated several of these residues of BicD2-CTD and performed pull-down assays with GST-tagged Nup358-min to assess the interaction and to confirm that these residues are important for the interaction with Nup358 but do not engage in an interaction with Rab6^GTP^. Notably, the BicD2-CTD mutants C745S, F750I, D755A, E756A, and D762A diminished binding to Nup358-min compared to the WT, but none of these mutants had a detectable effect on the interaction towards Rab6^GTP^ (Figure 9). These results confirm that the cargo interaction α-helix of Nup358-min indeed binds in the center of BicD2-CTD. This region of BicD2-CTD is expected to be remodeled by coiled-coil registry shift, which are a vertical displacement of the α-helices of the coiled coil by one helical turn. The identified BicD2 residues are specific for the interaction with Nup358-min and do not engage in the interaction with Rab6^GTP^. This is in line with our previous mutagenesis analysis of the Nup358 cargo-recognition α-helix. In this study, we mutated every residue of the Nup358 α-helix to alanine and assessed binding to BicD2-CTD by pull-down assays. Eleven interface residues were identified, confirming the interaction of the Nup358 cargo-recognition α-helix with BicD2 as the core interface of the complex. We conclude that the binding sites for Nup358-min and Rab6^GTP^ on BicD2-CTD are overlapping (BicD2 774–800 binds both Nup358 and Rab6) but distinct (BicD2 745–763 only binds Nup358). 

## 4. Discussion

Interactions between the dynein adapter BicD2 and its cargo Nup358 are important to activate and fine tune dynein motility for a nuclear positioning pathway that is essential for brain development [9]. Currently, a structure of a BicD2/cargo complex is not available, and therefore it is poorly understood how BicD2/Nup358 complexes modulate dynein motility. Here we propose a model for the Nup358/BicD2 complex based on structure prediction, docking, NMR results, and mutagenesis. Based on our results, Nup358 and BicD2 are oriented in the complex in an anti-parallel manner. The main stabilizing interaction of the complex is formed by the cargo-recognition α-helix of Nup358 [42], which binds to the center of the BicD2-CTD. We also identified a second interface between the intrinsically disordered N-terminal residues of Nup358-min which interact with the C-terminal ~50 residues of the BicD2-CTD. These ~50 C-terminal residues also bind to Rab6^GTP^, explaining why Rab6 competes with Nup358 for binding to BicD2 [27,29,36]. Our model provides a structural basis for the Nup358/BicD2 complex and will inform experiments that will establish how this interaction activates and finetunes dynein motility. 

Our results provide new insights into the cargo-recognition mechanism of BicD2. As previously established, the core of the Nup358/BicD2 interface is formed by the Nup358 cargo-recognition α-helix [42]. Here we show that this α-helix interacts with the central region of the BicD2-CTD, forming a helical bundle. Our structural model from Alphafold2 has a low predicted alignment error, suggesting a high confidence in the orientation of Nup358 respective to BicD2 in the model. The model is also supported by our NMR results and mutagenesis experiments. Our previous results showed that the interaction of the Nup358 cargo-recognition helix with the BicD2-CTD is an important modulator of dynein motility [42]. Mutations of the cargo-recognition α-helix reduced the formation of motile dynein/dynactin/BicD2/Nup358-min complexes on microtubules in single-molecule processivity assays and resulted in significantly reduced speed and run length of the reconstituted motor complexes [42]. This is not surprising since BicD2/cargo complexes are required for activation of dynein for processive motility; however, the underlying activation mechanism remains elusive. Future experiments will characterize cargo-induced structural changes in BicD2 that result in activation of dynein for processive motility. More specifically, we plan to test the hypothesis that the binding of Nup358 causes a coiled-coil registry shift in the BicD2-CTD (i.e., a vertical displacement of the α-helices against each other by ~one helical turn). Such registry shift would remodel the surface of the BicD2-CTD, thereby weakening the interaction with the N-terminal dynein-binding domain, activating BicD2 for dynein recruitment [28,29,42].

We also identified a second, weaker interacting domain that is formed by ten intrinsically disordered residues of Nup358 that are N-terminal of the cargo-recognition α-helix. This domain interacts with the C-terminal ~50 residues of the BicD2-CTD. While the Alphafold2 model itself has a higher PAE error for this region, our structural model of the Nup358/BicD2 complex is supported by mutagenesis and the NMR titration data which show that the ten N-terminal residues of Nup358-min interact with the BicD2-CTD. Therefore, the established contact regions between Nup358 and BicD2 are expected to be reliable, whereas some molecular details of the model for the N-terminal interaction domain may not be fully accurate. While this domain only contributes weakly to the overall Nup358/BicD2 interaction, it explains several previously made biological observations. It was previously shown that Rab6^GTP^, Nesprin-2, and Nup358 compete for binding to BicD2-CTD, and cannot bind at the same time [20,36]. The minimal domain of BicD2 required for binding of Nesprin-2 and Rab6^GTP^ is located in the C-terminal ~50 residues of the BicD2-CTD [20,27,29]. The cargo-recognition α-helix does not bind to this BicD2 region, so the second N-terminal interaction domain of Nup358 explains why it cannot bind to BicD2 at the same time as Rab6 and Nesprin-2 [20,36]. Furthermore, Nup358 has a 20-fold higher affinity to BicD2-CTD compared to Rab6^GTP^, which is in line with a larger interaction domain [36].

Nup358 is present in five copies per spoke on the cytoplasmic filaments of the nuclear pore. An oligomerization domain promotes an interaction of these five copies, but it is separated from the Nup358-min domain that interacts with BicD2 by long flexible linkers. It is therefore conceivable that the Nup358/BicD2 complex forms by simple association of the components, although the assembly mechanism remains to be established. It furthermore unknown how many motors are required for positioning of the nucleus [36]. There are 40 copies of Nup358 per NPC [55,56,57], and there are 2000 NPCs in G1 phase and 4000 NPCs at the onset of mitosis in human HeLa cells [36,58]. In G2 phase of the cell cycle, prior to entering mitosis, BicD2/dynein is recruited to the nuclear envelope by Nup358 [12]. Kinesin-1 actively positions the nucleus in G2 phase, fine tuning the motility in the Nup358/BicD2/dynein mediated nuclear positioning pathway [12]. This nuclear positioning pathway is regulated by phosphorylation of Nup358 through the G2-phase specific kinase cyclin dependent kinase 1 Cdk1, and potentially also by phosphorylation of BicD2 by Cdk1 and PLK1, which increases the binding affinity of BicD2 to Nup358 [2,59,60]. 

BicD2 is recruited to Nup358 at the cell nucleus in G2 phase of the cell cycle. Nup358 is phosphorylated by the G2-phase specific kinase cyclin dependent kinase 1 (Cdk1) at multiple sites (residues T2153, S2246, S2251, S2276, and S2280), which strengthens binding to BicD2 [2,59,60]. Four of these sites are the C-terminal of the Nup358-min domain. Some groups have suggested that Nup358 can only bind to full-length BicD2 after it is phosphorylated [59,60], and that phosphorylation of BicD2 by Cdk1 and Plk1 is also required for its recruitment to Nup358 at the nuclear envelope [60]. However, we would like to emphasize that phosphorylation is not required for this interaction. We have previously shown with single molecule binding assays that Nup358-min can bind efficiently to full-length BicD2 in the absence of phosphorylation [42]. However, the affinity of Nup358-min to full-length BicD2 is further increased by adding a C-terminal dimerization domain to Nup358-min, such as a leucine zipper [42]. It is conceivable that phosphorylation of Nup358 by Cdk1 promotes dimerization of Nup358, which would increase the affinity to BicD2. A second possible model is that a third interaction domain is formed between phosphorylated Nup358 residues and a BicD2 region that is N-terminal to the BicD2-CTD, which may increase the overall affinity. It would likely be separate from the Nup358/BicD2 interface characterized here, since our NMR titrations and mutagenesis data show that there are no interactions between Nup358-min and BicD2-CTD in the region that is C-terminal to the cargo-recognition α-helix. It should be noted that we obtained an Alphafold2 structure prediction of a larger complex with the entire coiled-coil 3 of BicD2 and a larger Nup358 domain that includes the Cdk1-phosphorylation sites (Figure S2). While the confidence scores of the added domains are low, interestingly, in the model, a third, separate interaction domain is formed by intrinsically disordered Nup358 residues 2256–2277, which include Cdk1-specific phosphorylation sites and BicD2 residues 690–733 (Figure S2). It is conceivable that such a third interacting domain is important for phospho-regulation of the interaction and future experiments will test this hypothesis.

Interestingly, while the binding sites for Nup358, Rab6, and Nesprin-2 on BicD2 are overlapping, the binding modes of each of these cargoes to BicD2 are slightly different, and therefore it will be important to establish structural models for all of these distinct BicD2/cargo complexes [20,42,49]. Several mutations in the cargo-binding domain of BicD2 cause devastating brain and muscle developmental diseases, including a subset of spinal muscular atrophy, which is the most common genetic cause of death in infants [20] Several of these point mutations affect binding of BicD2 to the cargoes Nup358, Nesprin-2 and Rab6 in a distinct manner, which may be the underlying molecular mechanism of these congenital diseases [20,49]. For example, the R694C mutation in human BicD2 causes a four-fold increase in binding to Nup358 and lowers the amount of Nesprin-2 recruited to BicD2 in cells by competition [20], resulting in defects in neuronal migration, for which the Nesprin-2/BicD2 nuclear positioning pathway is important [7]. The BicD2 mutations E774G and F743I/R747C in human Nup358 each strongly decrease binding to Nup358 and strongly increase binding to Nesprin-2, which resulted in defects in apical nuclear migration of brain progenitor cells, for which the nuclear positioning pathway facilitated by Nup358/BicD2 is essential [20]. Interestingly, the F743I and the R747C mutations both caused coiled-coil registry shifts in BicD2 in molecular dynamics simulations (i.e., a vertical displacement of the α-helices against each other by one helical turn) [28,49]. It is conceivable that some of these human disease mutations cause coiled-coil registry shifts in BicD2-CTD, which remodel its surface and result in changes in its cargo selection [20,28]. Our results suggest that understanding the molecular basis of the distinct BicD2/cargo interactions for each transport pathway is important in understanding the underlying molecular and structural mechanisms of diseases caused by BicD2 mutations. 

## 5. Conclusions

We present here a model of the structure of the Nup358/BicD2 complex, which provides insights into the cargo-recognition mechanism of BicD2. In the complex, the Nup358 cargo-recognition α-helix binds at the center of the BicD2-CTD. A second interaction domain is formed by 10 intrinsically disordered N-terminal residues of Nup358-min and the ~50 C-terminal residues of BicD2-CTD, which binds at the same site as the competing cargo Rab6, explaining why Rab6 and Nup358 cannot bind to BicD2 at the same time. Our structural model will be the basis for future experiments that will provide insights into how BicD2/cargo complexes activate dynein for processive motility and how the Nup358/BicD2 pathway for nuclear positioning is regulated by phosphorylation. It will also provide insights into how human disease mutations of BicD2 result in changes of cargo recognition and selection that may be the underlying structural mechanisms of devastating brain and muscle developmental diseases. 

## Figures and Tables

**Table 1:** Comparison of NMR results from Gibson et al. [42] with AlphaFold2 results.

Method	α-Helical Region	Binding Region
NMR	2162–2184 (CEST)	2149–2190 (titration)
AlphaFold2	2160–2186	2149–2191

**Table 2:** RMSD comparison of AlphaFold2 models with ClusPro and HADDOCK.

Docking Program	BicD2-CTD	Nup358-min
ClusPro	1.1 Å	0.34 Å
HADDOCK	0.95 Å	0.74 Å

## Data Availability

All data are either contained within the publication and supporting material or are available upon request from the corresponding authors.

## Supplementary Materials

The following supporting information can be downloaded at: https://www.mdpi.com/article/10.3390/biom13101445/s1, Figure S1: AlphaFold2 prediction models from ColabFold; Figure S2: AlphaFold2 prediction of a 2:2 complex of Nup358 (aa 2147–2290) and BicD2 (aa 634–804), which includes the known Cdk1-specific phosphorylation sites of Nup358 and the entire CC3 of BicD2; Table S1: Nup358/BicD2 Interacting Residues from Alphafold2; ClusPro Scoring Explanation; HADDOCK Scoring Explanation; LabeledGels.zip: Full original gel files with labels; ReplicateGels.zip: Replicate gels for quantitative analysis.
