# Supplementary material for: A Structural Model for the Core Nup358-BicD2 Interface
Source: Biomolecules. 2023 Sep 26;13(10):1445. doi: 10.3390/biom13101445 (PMC10604712; doi:10.3390/biom13101445)
Supplement: Supplementary file 1 [file biomolecules-13-01445-s001.zip › biomolecules-2568761-supplementary.pdf]

Supplementary information

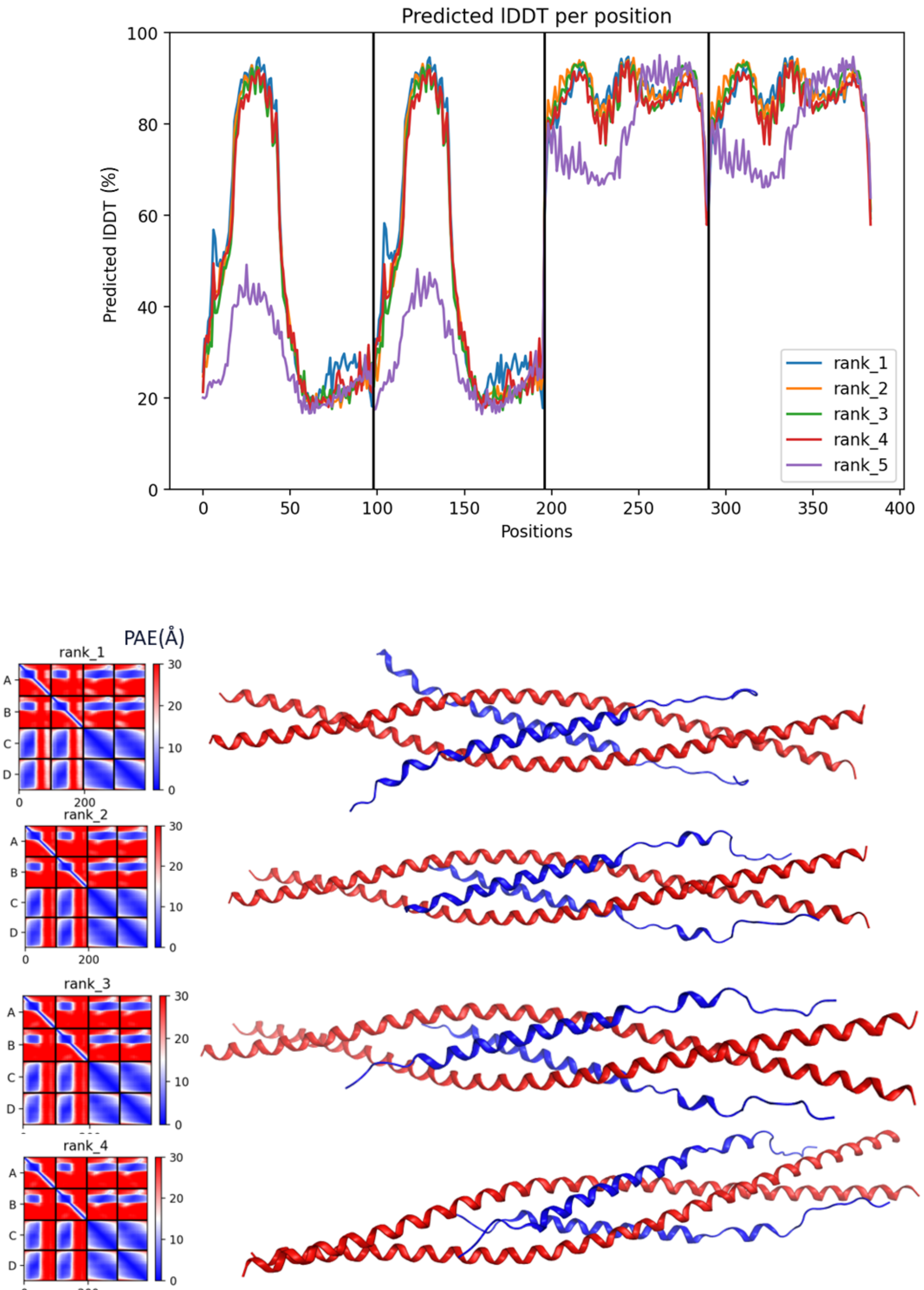

Figure S1: AlphaFold2 prediction models from ColabFold [51] Five models were provided with the first four models being similar in score, and the fifth model being much lower in score. The primary interface is the same in the first four models here as well as in the main model used in the publication. The secondary interface changes slightly but is present in all four models plus the main model in the paper. Further investigation was done with mutagenesis.

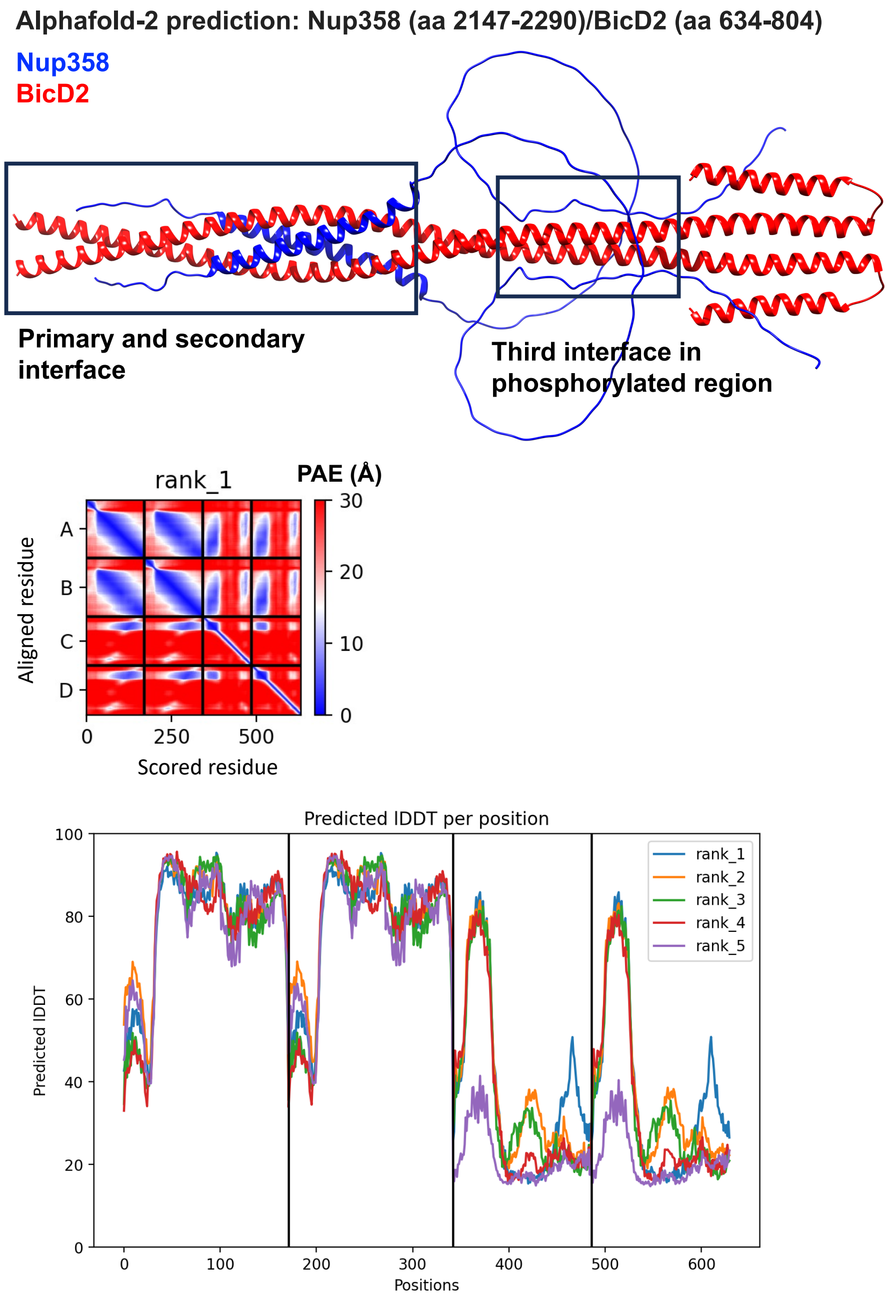

Figure S2: AlphaFold2 prediction of a 2:2 complex of Nup358 (aa 2147-2290) and BicD2 (aa 634-804), which includes the known Cdk1-specific phosphorylation sites of Nup358 and the entire CC3 of BicD2. The highest-ranking model is shown in cartoon representation (top panel). Note that a third interface is identified between BicD2 and Nup358 aa 2256-2277 which includes several Cdk1-specific phosphorylation sites[2]. This third interface is spatially separated from the core interface that is formed by the cargo recognition alpha-helix and the secondary interface (boxed). Middle panel: Predicted Aligned Error (PAE) plot for the highest ranked AlphaFold2 model. A color-gradient indicates the PAE in Å (0-10 Å: low error; 15 Å: acceptable threshold; 30 Å: low confidence). Bottom panel: Predicted per-residue local distance difference test pLDDT for five models. A pLDDT score of above 80% suggests confidence in the structure prediction (90-100%: high confidence; 80%: confidence threshold; 50%: low confidence). Note that the PAE and pLDDT scores of the additional BicD2 and Nup358 regions (i.e. additional to the Nup358-min and BicD2-CTD domains) that are included in this prediction are overall low.

Table S1: Nup358/BicD2 Interacting Residues from Alphafold2

Nup358 Interacting Residues	BicD2 Interacting Residues
I2149	E797
L2151	T793
K2156	K789
L2157	Q788
D2159	I786
T2160	R783
R2162	N779
A2163	K776
A2164	K775
L2166	E772
I2167	L768
R2168	Q767
A2170	Q765
E2171	M764
M2173	D762
K2174	L761
G2176	E760
L2177	I758
K2178	Y757
F2180	E756
K2181	D755
F2183	C754
L2184	R753
T2185	A751
D2187	F750
K2190	M749
V2191	R747
	L746
	S744
	F743
	A740
	D739

ClusPro Scoring:

The simulations in ClusPro resulted in 981 total models across 25 clusters, ranging in size from 1 at the low end to 199 at the high end. The weighted scores ranged from -645.9 to -772.3 for the center of each model and -652.6 to -922.3 for the lowest energy conformation in each cluster. The cluster with the highest number of entries (199) was consistent with that of the lowest energy conformation (-922.3).

HADDOCK Scoring:

The simulations in ClusPro resulted in 140 total models across 15 clusters, ranging in Z-scores from 1.3 to -2.2, and HADDOCK scores from -62.9 to -86.0. The lowest energy cluster had an RMSD from the lowest energy of 2.2 ± 1.3 Å.
